# In Colorectal Liver Metastases, the Presence of Extrahepatic Disease Correlates with the Pathology of the Primary Tumour

**DOI:** 10.5402/2011/948174

**Published:** 2011-06-22

**Authors:** Saleh Abbas, Vincent Lam

**Affiliations:** Westmead Hospital, Sydney, NSW 2145, Australia

## Abstract

*Background*. FDG-PET scan detects extrahepatic metastases in 20% of patients with colorectal liver metastases but it is reported to have approximately 16% false negative rates. *Patients and Methods*. Patients who had PET scan for metastatic colorectal cancer at Westmead Hospital between March 2006 and March 2010 were reviewed retrospectively. The results of PET scan were correlated with tumour characteristics that were thought to affect the overall prognosis. *Results*. Degree of tumour differentiation and vascular invasion were significantly predictive for the presence of extrahepatic disease on PET scan, also did the level of CEA. *Conclusion*. The detection of extrahepatic disease in colorectal liver metastases correlates with the biologic behaviour of the primary tumour. Poorly differentiated tumours and those with lymphovascular invasion behave in aggressive fashion and likely to have wide-spread metastases. This should be considered when contemplating liver resection for colorectal metastases.

## 1. Introduction

Liver resection is currently the most effective treatment for patients with colorectal cancers metastatic to the liver. Stringent selection policy is necessary because there is no survival benefit if residual disease is left elsewhere after hepatectomy [1].

FDG-PET scan detects extrahepatic metastases in 20% of patients with colorectal liver metastases but it is reported to have approximately 16% false negative rates [2]. Positron emission tomography with the glucose analog [^18^F] fluoro-2-deoxy-D-glucose is a sensitive diagnostic test that detects tumours based on the increased metabolic utilisation of glucose by tumour cells. FDG-PET has been demonstrated to be more sensitive than CT in the detection of metastatic colorectal adenocarcinoma, particularly in extrahepatic locations [3].

 Positron emission tomography provides useful information in the selection of patients with hepatic metastases from colorectal cancer being considered for surgical therapy; it alters the decision of management in 15% of such patients, which helps to reduce the number of unnecessary surgical explorations and results indirectly in improved survival in patients undergoing hepatic resection [4]. Positron emission tomography is currently being routinely integrated in preoperative evaluation of patients being considered for liver resection of colorectal metastases.

The overall sensitivity of 97% and an overall specificity of 76% for FDG-PET in detecting recurrent colorectal cancer were documented by one systematic review and meta-analysis of studies conducted to investigate the role of PET in colorectal liver metastases [5]. 

Fernandez et al. reviewed the literature for five-year survival after liver resection for colorectal metastases before the introduction of PET scan used to be average of 30% (12–40%). With the routine use of PET scan the actuarial five-years survival has improved to up to 58% [3].

Previous studies found some correlation between certain clinical indicators such as interval for hepatic recurrence, size of liver metastases, and CEA levels and the results of PET scan for detection of extrahepatic metastases [2].

Tumour biology as reflected by degree of differentiation, nodal metastases, T stage, and possibly other biologic markers is found to affect the overall survival after liver resection for metastatic colorectal cancer [6]. 

We designed this study to estimate the correlation between clinical factors related to the diseases and pathological features of the primary colorectal cancer with the results of PET scan for patients presented with liver metastases and being considered for liver resection. 

## 2. Patients and Methods

The database for PET scan records was searched for patients who had PET scan with history of colorectal cancer and their imaging showed hepatic metastases. Patients were identified and their medical records were reviewed. Patients who proved to have liver metastases on PET scan were included in this study for the period between March 2006 and March 2010.

Data collected include demographics and interval between colorectal cancer resection and the appearance of liver metastases; results of the PET scan were recorded and included presence of extrahepatic disease, number and size of liver metastases, and bilobar liver disease; conventional cross-sectional imaging data were recorded. We also collected data from operative reports (including the findings on intraoperative ultrasound) and perioperative records.

The pathology report of the primary colorectal cancer was reviewed, and data extracted included site of the primary disease, degree of differentiation, lymphovascular invasion, T stage, N stage, and apical nodes involvement. Factors thought to correlate with the presence of extrahepatic disease were analysed using chi-squared test. Multivariate analysis was performed using logistic regression test for independent factors that correlate with extrahepatic disease detection as found on PET scan. 

## 3. Results

Between 2006 and March 2010, 230 PET scans were performed; 174 patients with history or newly diagnosed with colorectal cancer had PET scan for evaluation of suspected liver metastases; the rest of the patients either had rising CEA or suspected extrahepatic metastases. These patients had suspicious liver lesions on CT scan and/or rising CEA levels. 106 patients (47 women and 59 men) with median age of 63 (range 26–88) proved to have liver metastases with or without extrahepatic recurrence.

The site of primary lesion was in the right colon in 25 patients, rectum in 20 patients, left colon and sigmoid in 56 patients, and transverse colon in 5 patients. All patients had CT scan of the chest, abdomen, and pelvis.

Liver metastases were synchronous in 30 patients, 37 patients had recurrence within 12 months of resection of the colorectal cancer, and 39 patients had recurrence after 12 months (range 14–72 months). PET scan was false-negative in three patients who had CT evidence of liver disease and proceeded to have liver resection, which proved the metastatic nature of the lesion that was seen on cross-sectional imaging. PET scan detected lesions that were not seen on CT scan in 12 patients, eight of those were extrahepatic lesions and four were liver lesions.

Pathology of the primary colorectal cancer was moderately differentiated in 72 patients (68%), poorly differentiated in 30 patients (28%), and well differentiated in 4 patients (4%). Lymphovascular invasion was absent in 70 patients (66%) and was seen in 36 patients (34%). The primary lesion was T2 in 14 patients, T3 in 72 patients, and T4 in 20 patients. The disease was N0 in 23 patients and N2 in 83 patients. Apical lymph nodes were involved in 11 patients.

There was one liver lesion in 47 patients, 19 patients had two lesions, 9 patients had three lesions, 8 patients had 4 liver lesions, and 23 patients had five or more lesions. The disease was bilobar in 27 patients.

CEA level was normal in 18 patients, and serum levels ranged between 0.5 mcg/L and 16223 mcg/L.

PET scan showed that the disease was limited to the liver in 65 patients and 41 patients had extrahepatic disease. The most frequent sites were lungs, peritoneal disease, local recurrence, mediastinal nodes, and occasionally bones and brain. 

### 3.1. Statistical Analysis

Data were analysed using StatsDirect. Univariate analysis of factors that may correlate with the presence of extrahepatic disease showed that lymphovascular invasion, degree of differentiation, T stage, CEA level, size of metastases, and bilobar disease were significant predictors of the presence of extrahepatic disease (see Table 1).

Multivariate analysis was performed using stepwise logistic regression test showing that vascular invasion, degree of differentiation, and CEA level are the only independent factors that predict the presence of extrahepatic disease (see Table 2).

## 4. Discussion

Cross-sectional radiologic imaging utilizing abdominal and pelvic CT scan is the most commonly used imaging to evaluate liver anatomy and assess for the presence of extrahepatic disease in patients with colorectal liver metastases who are potential candidates for curative liver resection. 

PET scan was found in other studies to reduce the rate of nontherapeutic laparotomy from 15% to less than 5% [7]. The same investigators from John Hopkins Center tumor number greater than five, bilateral liver disease and tumor size larger than 5 cm were independent factors associated with presence of extrahepatic disease. Huebner et al. conducted a meta-analysis of 11 articles of the use of PET scan for colorectal liver metastases; they determined, an overall sensitivity of 97% (95% confidence level: 95–99%) and an overall specificity of 76% (95% confidence level, 64–88%) for FDG PET detecting recurrent CRC throughout the whole body. Furthermore, through pooling of the change-in-management data, an overall FDG PET-directed change in management was calculated to be 29% (95% confidence level: 25–34%) [5].

The present study is a retrospective review of patients referred to Westmead Hospital for assessment of colorectal liver metastases to confirm the extent of liver disease and to assess for the presence or absence of extrahepatic disease; due to the pattern of referral we were able to review only patients who had their medical record at Westmead Hospital. The subjects of this study were patients who proved to have liver metastases on cross-sectional imaging and PET scan. We correlated clinical factors and pathology of the primary colorectal cancer with the finding of the PET scan. On multivariate analysis we found that presence of vascular invasion, degree of differentiation of the primary lesion, and CEA level are the only factors associated with the presence or absence of extrahepatic disease. While tumour stage of the primary and nodal metastases, time interval between the diagnosis of the primary and the recurrence, and size and number of metastases in the liver were significant predictors on univariate analysis; they were not significant factors in the multivariate analysis. Lymphovascular invasion seems to be a very significant factor in tumour biology; patients with primary tumor showing lymphovascular invasion would have a significant higher chance of lymph node metastasis. Positive lymph node status was predictive of poorer survival in patients with T1 or T2 colorectal cancers. For those cancers with positive lymphovascular permeation, radical surgery is recommended [8].

Lim et al. studied 2417 patients; a lymphovascular invasion-positive tumor was detected in (25.2%). Compared with patients with lymphovascular invasion-negative tumors, those with lymphovascular invasion-positive tumors had higher preoperative serum carcinoembryonic antigen levels. Their tumors were also more likely to be poorly differentiated and more advanced in terms of T and N categories. The lymphovascular invasion-positive tumors were also more likely to have metastasized systemically. Lymphovascular invasion-positive tumors metastasized to systemic lymph nodes more often. These tumors also recurred at systemic lymph nodes after curative intent surgery more often. Lymphovascular invasion-positive status was an independent unfavorable prognostic factor for the 5-year overall and 5-year disease-free survival of patients with sporadic colorectal cancer [9].

Pawlik et al. described factors associated with nontherapeutic laparotomy for colorectal liver metastases, which included size of metastases, number of metastases, and bilateral disease [7]; however, they did not include factors related to the primary cancer pathology. Inclusion of tumour-related factors in our study has shown that liver lesion factors are important determinants on their own but were not significant in multivariate analysis. Disease-free interval was shown in some previous studies to be an important factor that predicts extrahepatic disease [2]. In our study and the study by Pawlik et al. [7], time interval was not a significant factor in prediction of behaviour of colorectal cancer spread.

It has been described that overall survival for patients undergoing curative resection for colorectal liver metastases can be predicted from the pathologic features of the primary colorectal cancer [6] depending on the degree of differentiation of the primary cancer and presence or absence of lymph nodes metastases. Survival after resection of colorectal liver metastases is continually improving due to better patients' selection, improved postoperative care, and more effective chemotherapy agents [10].

Das et al. have investigated the significance of pathologic and biology features of primary rectal cancer and concluded that pathologic T and N stages significantly predicted overall survival, distant metastases, and loco regional recurrence on multivariate analysis and that indirectly is an indication for investigations of more aggressive adjuvant chemotherapy for locally advanced rectal. More recent aggressive chemotherapy and molecular therapy had shown significant improvement of overall survival for metastatic colorectal cancer [11, 12]. In another study, Harris et al. investigated factors that affect survival in patients present with advanced metastatic colorectal cancer and, on multivariate analysis, factors that had impact on survival included, T stage, N stage, and degree of differentiation of the primary disease [13]. In patients with locally advanced rectal cancer who underwent total mesorectal excision the independent factors for poor survival were the advanced stage of the disease and the presence of lymphovascular and perineural invasion [14]. In patients with Duke's B stage the presence of lymphovascular invasion is associated with increase rate of disease recurrence, and hence this feature is used as an indication for chemotherapy after curative resection [15, 16].

Biology of the primary tumor is increasingly being recognized as a primary determinant factor in disease behavior and overall outcome; one such factor is perineural invasion (PNI): in one study it was detected in 57 of 341 patients (16.7%) and was significantly associated with depth of tumor invasion and positive lymphovascular invasion. Multivariate analyses revealed that PNI was a significant independent prognostic factor for disease-free survival, not for overall survival [17]. More emphasis on the biology of the primary tumor has been focused on the genetic features of the primary tumor in one study of primary colorectal cancer; p53 mutations were a significant negative prognostic indicator for overall survival. This finding holds prognostic and therapeutic implications for the management of colorectal carcinoma patients [18, 19]. Future investigations may reveal more molecular markers that not only may predict prognosis but also will have implications of investigation and treatment of metastatic disease, which may dictate which subset of patients with liver metastases should undergo PET scan imaging.

Biology of liver metastases tends to follow the features of the primary disease. Rajaganeshan et al. studied this subject and found that primary cancers with a high microvaswcular density (MVD) tended to form high MVD liver metastases. Microvessel density was a significant predictor of disease recurrence in Primary tumour tumours. These results suggest that primary CRCs and their liver metastases show common histological features [20]. And those tumours with high microvascular density have high risk for recurrence [21].

In conclusion, biological factors related to the primary tumours such as vascular invasion, degree of differentiation, p53 mutation, microvascular density, and perhaps new molecular agents may be useful in the future to guide the choice for the need of PET scan for colorectal metastases and may dictate the type of therapy each subset of patients should be subjected to. 

## Figures and Tables

**Table 1:** Results of univariate analysis.

	No extrahepatic disease on PET	PET positive for extrahepatic disease	*P*
Age			
Less than 60 years	17	23	.6
Over 60 years	24	42	
Interval for recurrence			
Less than 12 months	31	37	.08
More than 12 months	10	28	
Degree of differentiation of primary tumour			
Well moderate	59	18	<.0001
Poor	6	23	
Lymphovascular invasion			
Present	61	9	<.0001
Absent	4	32	
T stage			
T2	12	2	.02
T3-4	53	39	
N stage			
N0	16	7	.4
N1	49	34	
CEA level			
Less than 20 mcg/L	41	12	.001
More than 20 mcg/L	24	27	
Size of the liver metastases			
Less than 5 cm	47	21	.02
5 cm or more	18	20	
Bilobar Disease			.001
Yes	7	21	
No	58	20	

**Table 2:** 

diff	*P* = .0046	
vascular	*P* < .0001	
T	*P* = .8301	
Size	*P* = .3677	
Bilobar	*P* = .3571	
CEA	*P* = .024	

Logistic regression-odds ratios		
Parameter	Odds ratio	95 CI

diff	11.536075	2.12326 to 62.67768
vascular	41.720557	8.388554 to 207.497598
T	1.145417	0.331347 to 3.959542
Size	0.472701	0.092598 to 2.413074
Bilobar	2.327326	0.385553 to 14.048527
CEA	6.23202	1.5 to 3.7
